# Supporting Cancer Patients in Illness Management: Usability Evaluation of a Mobile App

**DOI:** 10.2196/mhealth.3359

**Published:** 2014-08-13

**Authors:** Jelena Mirkovic, David R Kaufman, Cornelia M Ruland

**Affiliations:** ^1^Center for Shared Decision Making and Collaborative Care ResearchOslo University HospitalOsloNorway; ^2^Department of Biomedical InformaticsCollege of Health SolutionsArizona State UniversityScottsdale, AZUnited States

**Keywords:** mobile applications, patients, cell phone, smartphone, symptom assessment, self-care, user-computer interface

## Abstract

**Background:**

Mobile phones and tablets currently represent a significant presence in people’s everyday lives. They enable access to different information and services independent of current place and time. Such widespread connectivity offers significant potential in different app areas including health care.

**Objective:**

Our goal was to evaluate the usability of the Connect Mobile app. The mobile app enables mobile access to the Connect system, an online system that supports cancer patients in managing health-related issues. Along with symptom management, the system promotes better patient-provider communication, collaboration, and shared decision making. The Connect Mobile app enables access to the Connect system over both mobile phones and tablets.

**Methods:**

The study consisted of usability tests of a high fidelity prototype with 7 cancer patients where the objectives were to identify existing design and functionality issues and to provide patients with a real look-and-feel of the mobile system. In addition, we conducted semistructured interviews to obtain participants’ feedback about app usefulness, identify the need for new system features and design requirements, and measure the acceptance of the mobile app and its features within everyday health management.

**Results:**

The study revealed a total of 27 design issues (13 for mobile apps and 14 for tablet apps), which were mapped to source events (ie, errors, requests for help, participants' concurrent feedback, and moderator observation). We also applied usability heuristics to identify violations of usability principles. The majority of violations were related to enabling ease of input, screen readability, and glanceability (15 issues), as well as supporting an appropriate match between systems and the real world (7 issues) and consistent mapping of system functions and interactions (4 issues). Feedback from participants also showed the cancer patients’ requirements for support systems and how these needs are influenced by different context-related factors, such as type of access terminal (eg, desktop computer, tablet, mobile phone) and phases of illness. Based on the observed results, we proposed design and functionality recommendations that can be used for the development of mobile apps for cancer patients to support their health management process.

**Conclusions:**

Understanding and addressing users’ requirements is one of the main prerequisites for developing useful and effective technology-based health interventions. The results of this study outline different user requirements related to the design of the mobile patient support app for cancer patients. The results will be used in the iterative development of the Connect Mobile app and can also inform other developers and researchers in development, integration, and evaluation of mobile health apps and services that support cancer patients in managing their health-related issues.

## Introduction

### Mobile Health Apps—Opportunities and Challenges

Mobile devices are continuously present in people’s everyday lives [1], and many individuals have a deeply personal relationship with their mobile phones, which are typically customized to their specific needs [2,3]. Evolving technical capabilities of mobile devices enable delivery of various services independent of the user’s time and place, and their dynamic adaptation to current context of use and users’ personal preferences [4]. These features make mobile devices well-suited terminals for easier monitoring and managing of pre-existing health conditions, the delivery of more efficient, individually tailored care at the point-of-need, and promotion of better collaborative work between patients and health care providers [5-10].

However, mobile devices’ hardware limitations (eg, small screen, limited input capabilities) introduce numerous challenges when migrating from an existing eHealth Web-based system to a mobile platform. Some of the general guidelines are to (1) provide support only to a limited number of features to eliminate the variety of options that are not core to the mobile use case, (2) show only limited content to reduce word count and provide better visibility and glanceability, and (3) enlarge interface elements to enable easier input of data and accommodate the “fat finger” problem [11,12]. Research has explored methods of automating the migration process from Web-based to mobile-based systems (eg, [13,14]), but such work has mainly focused on increasing efficiency of translation techniques, rather than identifying system requirements in the new context. When designing and developing mobile health apps, special care must be taken to address patients’ specific needs, which often vary greatly across different contexts beyond type of access terminal (eg, type of illness and diagnosis, phase in treatment, and patient demographics and literacy). For example, literacy, health literacy, and previous experience with technology can significantly influence patients’ usage and navigation through (mobile) health apps and their ability to apply the knowledge gained to managing health conditions [15,16]. Also, the granularity of data that is captured on the mobile device and used for monitoring of health conditions and behaviors is highly dependent on the patient’s condition. While a simplified 5-point scale describing the size of the meal is good enough for logging food intake of persons who are trying to get in shape and lose weight, it is not sufficient for diabetes patients who need to carefully monitor relation of food intake and blood glucose level [7]. Another example includes the use of metaphors and graphical representations to show the user’s progress towards some predefined goal or current (health) status. While metaphors on glanceable displays are shown as highly effective for maintaining and increasing the physical activity of users [8,17], their use for presenting the status of more serious health-related conditions are not well accepted by patients (eg, neutral graph visualization metaphors were found to fit better for patients with mental illness [18]).

Previous research has also shown that advanced age and lack of experience with mobile technology decreases peoples’ ability to create accurate and useful spatial mental models of a mobile app’s menu and navigation structure [19]. A mental model is defined in cognitive psychology as a user’s internal representation of an external system’s structure and functions [20]. Mental models are usually formed by combining previous knowledge and experience with similar systems, cognitive schemes, and problem-solving strategies [21]*.* The absence of an accurate mental model can significantly influence a user’s task performance on mobile devices and can lead to disorientation in menu selection [19,22].

In system development, the user-centered design (UCD) approach is used to identify and address end-user requirements and adjust (mobile) system design and functionality to user’s capabilities, needs, and expectations [23-26]. UCD incorporates a range of methods ranging from focus groups to iterative usability testing and participatory design [27]*.* Applying UCD principles to development of mobile health information services for patients can support users in changing their health-related behavior [26,28].

### Mobile Apps for Cancer Patients

Cancer patients often experience a wide range of physical, functional, and psychological symptoms during treatment and rehabilitation. Failure to identify and address these symptoms during hospital admissions can lead to considerable distress [29,30]. Also, the side effects of treatments usually cause a range of new symptoms that are often worse after patients have been discharged from the hospital. Therefore, Web-based systems that can support management of symptoms and health care-related issues at home could be beneficial for this patient group [31]. However, few projects can be found in the literature that address the design and implementation challenges of mobile information services intended to support cancer patients in managing their illness and health-related issues. For example, Leimeister et al researched how standard features of personal digital assistants—such as diaries, SMS (short-message service), email, messaging—can be used to support adolescent cancer patients in illness management [32]. The results showed that using the mobile devices helped patients in coordinating their extensive treatment schedules and medication plans. To achieve this goal, the calendar function was found especially useful. The diary function was not well accepted due to the limitation of a small keyboard. Communication features such as SMS and email messages were well accepted but mostly for communication with family members and friends rather than health care personnel [32].

Klasnja et al described the design of HealthWeaver Mobile, an app that helps patients manage care-related information during treatment [33,34]. The study outlines the general design and functionality requirements for developing mobile systems that target cancer patients, such as the ability to install the mobile app on a personal phone, the importance of standard integrated features (eg, camera, microphone), and useful system functionalities (eg, calendar, notes, registration). The mobile app is developed as part of a greater symptom-management system that also includes a Web app. The authors argued that the use of a native Web hybrid approach offered a more cost-effective way to provide cross-platform support in mobile health tools.

The ASyMS system supports remote monitoring and symptom management of chemotherapy-related symptoms in cancer patients [35,36]. Using their mobile phones, patients fill out and send a report with their symptoms and then immediately receive feedback consisting of tailored self-care advice directly related to the severity level of the symptoms they just reported. An additional evidence-based risk assessment tool alerts clinicians when an incoming patient-reported symptom is considered critically important. A randomized control trial showed that the mobile app can provide valuable support to chemotherapy patients for symptom management and improve patient-provider communication. The feasibility of a similar system for monitoring chemotherapy-related issues is reported in [37], demonstrating the benefits of real-time telemedicine with remote nurse support.

A Wireless Health Outcomes Monitoring System (WHOMS) and the electronic Edmonton Symptom Assessment System (e-ESAS) are additional examples of mobile systems that provide remote monitoring of cancer patients’ health issues by health care providers. In WHOMS [38], the medical management team sends structured questionnaires to the patient’s mobile phone, and the completed questionnaire is then reviewed by the medical team. The e-ESAS system [39] was developed and adapted for use in developing countries to enable patients to easily report symptoms, as well as to enable palliative doctors to preview and process data.

In summary, research has addressed a range of issues related to the design and development of a mobile information system that enables the remote monitoring of cancer patients by health care providers while also supporting patient’s self-management of health-related issues and preparation for clinic consultations. The evolution of social media and advances within communication technology provide new opportunities for patients to become more engaged in different types of discussion and reflections regarding their own health issues. The importance of these functionalities is discussed in previous research [7,32,40,41]. However, to our knowledge there is no research addressing issues regarding design and development of mobile app(s) for cancer patients that support a wider range of system features including advanced online communication among patients and between a patient’s health care provider as well as symptom management tools.

To address issues related to design and development of mobile apps for cancer patients that provide a greater variety of system features (both symptom management and communication), we present the design, development, and evaluation of the Connect Mobile app. The Connect Mobile app is part of the previously developed and deployed Connect system, which provides support to cancer patients in managing health-related issues and promotes better patient-provider communication, collaboration, and shared decision making. The goal of this work was to (1) identify design challenges and issues related to providing mobile access to a patient support system such as the Connect system, (2) evaluate perceived usefulness and user acceptance of the system and its features across different access terminals, (3) investigate the new context of use and new system requirements introduced by enabling mobile access to the patient support system, and (4) contribute to a robust and extensible mobile app design framework for patients with chronic illness.

### The Connect System

**Figure 1:**
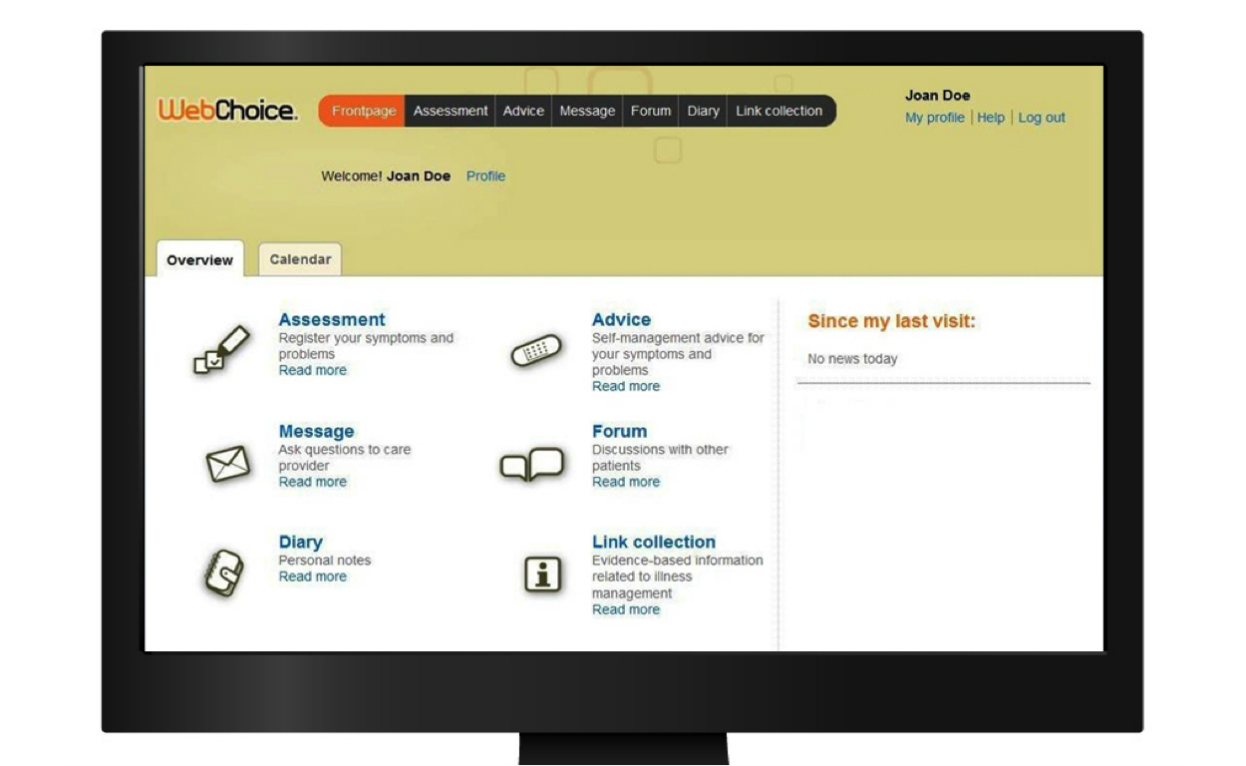
Connect Web app (screenshot of the main menu).

The work reported in this paper is part of the Connect research project. Its goal is to promote timely, secure, and seamless collaboration between chronically ill patients and health care providers on different levels of care by using a device-independent, mobile, and multifunctional Internet platform called Connect (formerly known as WebChoice). The Connect system and its components were developed based on patient-centered principles and designed to support cancer patients in self-managing their illnesses and to enhance patient-centered care. The Connect system’s design and functionalities were developed in cooperation with system stakeholders (ie, patients, health care providers) using numerous user-centered and participatory design methods (eg, focus groups, usability evaluation, heuristic evaluation) [31,42,43]. The system incorporates a series of modules designed to support patient-provider communication, collaboration, and shared decision making in different environments (eg, hospital, outpatient clinic, and patients’ homes). Patients can access the Connect system through a Web browser on their personal computers or laptops (Figure 1) to (1) report and monitor their symptoms and health problems by selecting from predefined categories and rate their level of distress and priority for support (assessment module), (2) obtain individually tailored evidence-based self-management support (symptom self-management support module), (3) get access to other reliable Internet resources (information module), (4) ask questions and receive advice and professional support (messaging module), (5) share and discuss their experiences with other patients (communication module), and (6) note their private health-related information as free text (diary module).

The Connect system was tested in a randomized clinical trial with 325 breast- and prostate-cancer patients from all over Norway. The results showed that the system improved patient-provider communication, decreased symptom distress and depression, and provided better self-efficiency for patients [31,42,44]. Additionally, system logs analysis showed that all system components were used independently of users’ diagnosis type, stage of disease, age, or previous computer experience [41]. However, usage patterns differed for patient subgroups. For example, even though the functions that enabled patient-patient and patient-provider communication were used the most frequently, in general breast-cancer patients used the system more for seeking health-related information while prostate cancer patients focused more on features that helped them to prepare to talk with health personnel. Patients with a long-term illness history were more active in communication (both patient-patient and patient-provider) and exhibited more information-seeking behaviors than patients who received a diagnosis for the first time [41,45]. Additionally, messaging and symptom self-management support functionalities were used more by patients with a low level of social support and a high level of symptom distress and depression [40]. The general conclusion was that there is no “one size fits all” system, and user preferences and use patterns are dependent on numerous factors including personal characteristics, illness type, disease stage, social support, and illness burden.

Since the Connect system provides a device-independent, multifunctional Internet platform, in the current research project we investigated how the system’s functions could be further translated to new contexts of use by enabling access over mobile devices such as mobile phones (which refers to smartphones but not regular cell phones) and tablet computers. The work presented in this paper addresses different design and implementation challenges that surfaced during the development and evaluation of Connect Mobile, a mobile app that enables access to the Connect system. The initial version of the Connect Mobile app design was developed to adhere to general and widely accepted usability guidelines for different types of mobile devices (eg, design guidelines from device and software manufacturers [46,47] and general guidelines for development of user-friendly mobile apps and interfaces [12,48]). Since basic design rules for development of a mobile app underline the importance of simplicity due to device limitations (eg, small displays and limited input characteristics), the Connect Mobile app was implemented to allow access to only a subset of all functionalities offered by the Connect system. Based on previous research on the Connect project that identified patients’ usage patterns and functional requirements, we endeavored to incorporate the following modules and features in the Connect Mobile app:

- The messaging module where patients can exchange messages with health care personnel (eg, primary and specialist care physicians and nurses).
- The assessment module where patients can record and keep track of problems that are bothering them. The symptoms assessment module requires the following four steps: (1) patients select the symptoms that are bothering them from the list of predefined symptoms organized in groups and subgroups, (2) for each selected symptom, the patient selects how bothersome it is, (3) the patient selects how important it is to address the symptom in the meeting with health provider, and (4) before submitting the report, the patient can review the list and go back to previous steps and perform changes if needed. The mobile app also gives the patient an option to see the list of previously reported symptoms.
- The symptom self-management support module where patients can get information about managing self-reported symptoms, how to psychologically deal with the illness, the challenges it brings, and their rights as a patient. Patients can also create a personalized list of advice and activities that they find most useful and valuable.
- The communication module/forum functionality where a patient can exchange information and experiences with other patients using the Connect system.

The Connect Mobile app was developed using PhoneGap, an opensource framework for building cross-platform mobile apps [49]*.* The PhoneGap platform supports development of a single mobile app using standard Web-based technologies (such as HTML, cascading style sheet, and JavaScript), which can run as a native app on various mobile platforms and operating systems (such as Android, iOS, Windows Phone). We chose the development of a native app because they are better accepted by end users than Web pages and provide better support for customization to device characteristics [50]. Additionally, the native apps can make use of the mobile devices’ features (eg, camera, positioning).

For implementation of the mobile app design, we used JQuery Mobile framework, which provides support for development of HTML5-based user interfaces for all popular mobile device platforms as well as a library of standard and widely accepted mobile app widgets and interface elements. We applied a responsive Web design approach that supports dynamic adaptation of app interface design to a device’s characteristics (screen size, platform type, device orientation), while the app logic was the same for both types of devices (mobile phones and tablet computers). For example, while the bottom tabs are used to enable transition between features on mobile phones, the left side menu was used for the same type of navigation on tablet devices. Additionally, the design of interface elements (eg, size, order, positioning on the screen) was adjusted to fit characteristics of these two types of devices. The communication with underlying Connect system uses hypertext transfer protocol and follows representational state transfer architecture guidelines for designing networked apps.

**Figure 2:**
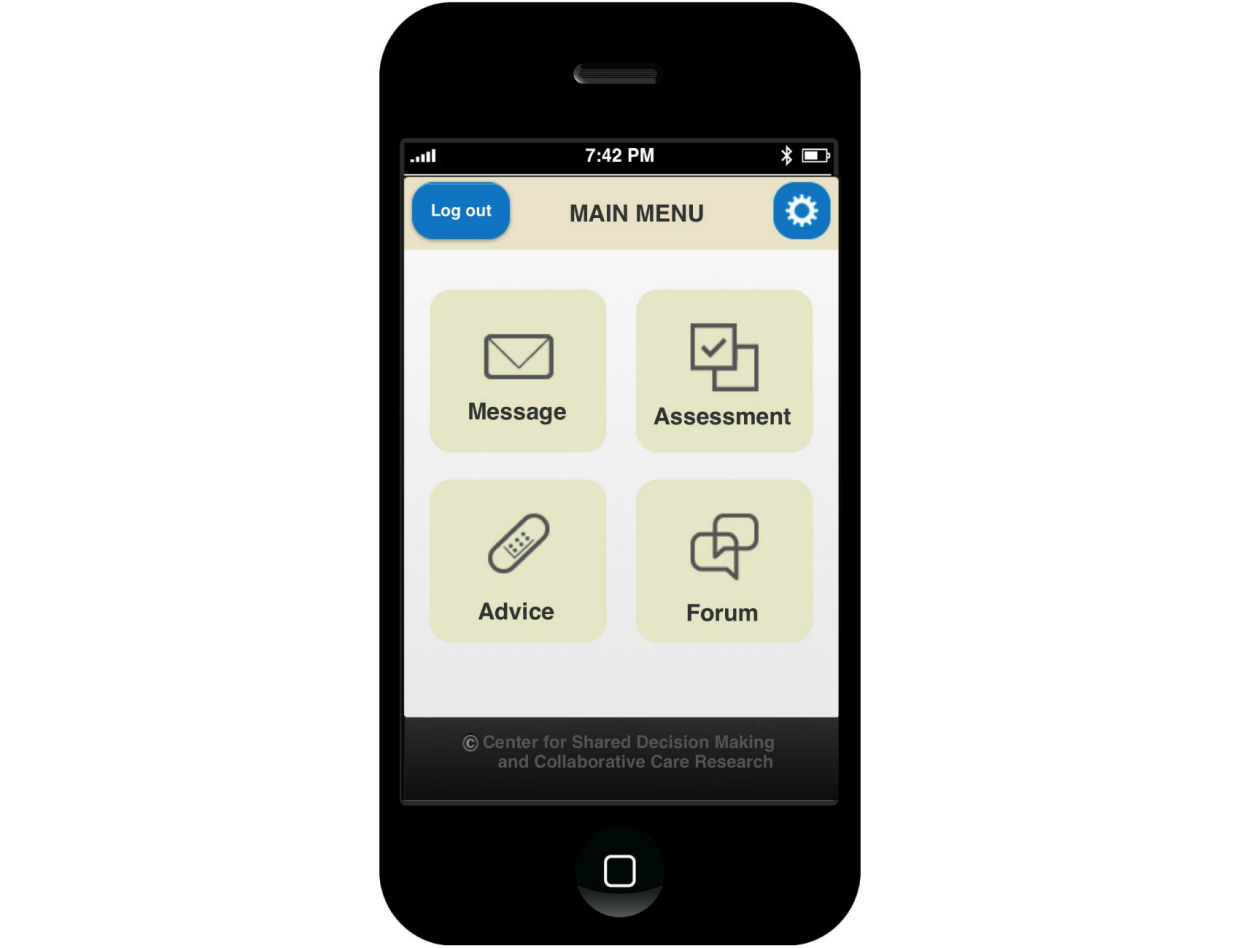
Connect smartphone app (screenshot of the main menu).

**Figure 3:**
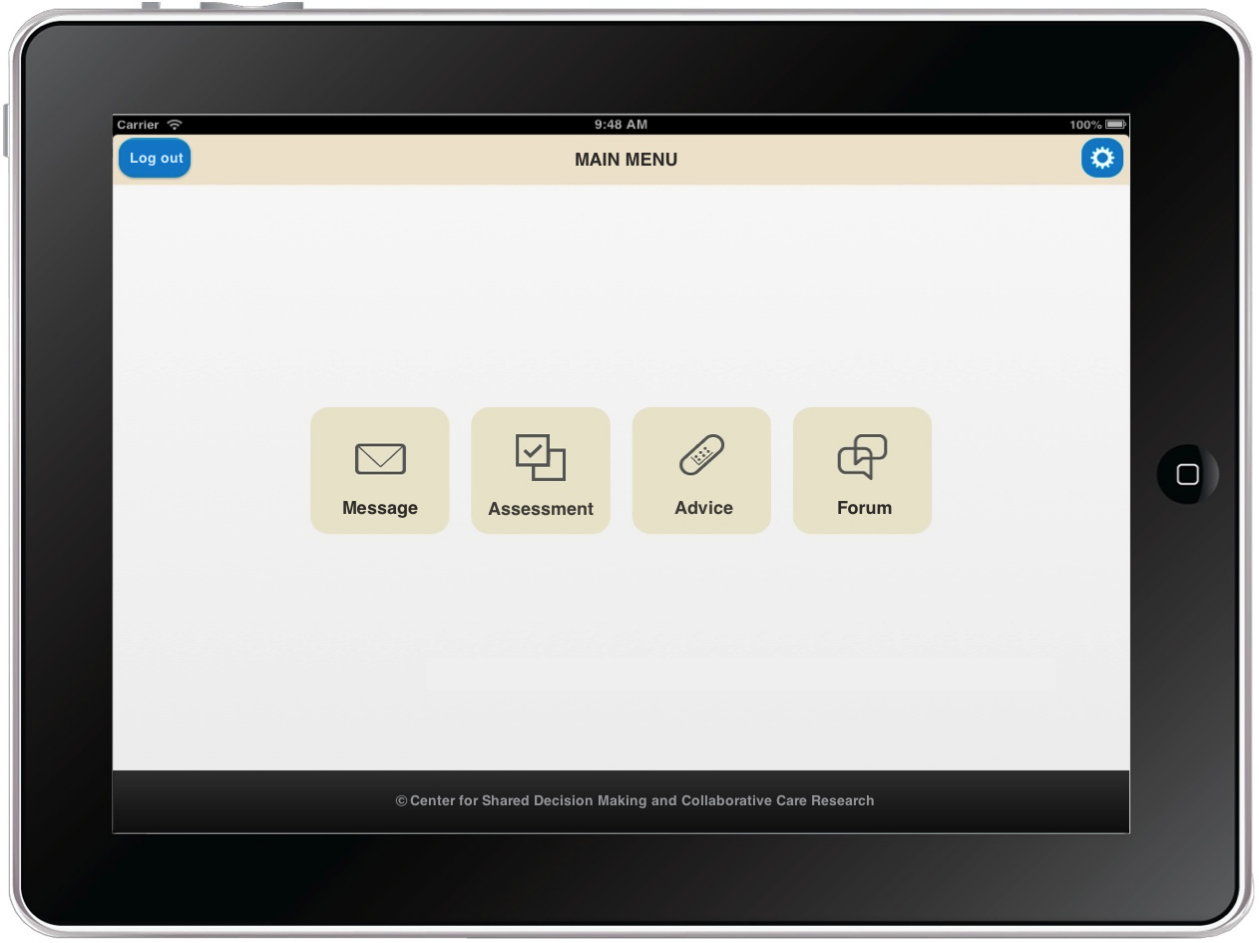
Connect tablet app (screenshot of the main menu).

A preview of Connect system main menu interface is shown on different access terminals in Figures 1-3.

## Methods

To evaluate the Connect Mobile app, we performed a usability evaluation study of both the mobile phone and tablet computer versions of the app. The study consisted of usability testing of a high fidelity prototype where our objectives were to identify existing design and functionality issues along with usability problems and to provide patients with a real look-and-feel of the mobile system. In addition, we conducted semistructured interviews to solicit responses from participants about app usefulness, identify the need for new system features and design requirements introduced by the new context of use, and determine the acceptance of the mobile app and its features in everyday health management.

Our study sample consisted of 7 cancer patients from a rural municipality in the northern part of Norway. Inclusion criteria were that patients were participants in the larger study where they were given access to the Connect system on their private computers, and that they had previous experience using the system.

The usability study was conducted on two devices: (1) an iPod device that simulated a mobile phone with access to a wireless network and (2) an Asus Transformer tablet computer. The devices were tested as follows: 2 participants tested the mobile phone version of the app, 3 participants tested tablet PC version, and 2 participants tested both versions. Since the 2 participants were tested in separate sessions separated by 3 months, we concluded that first testing session did not considerably affect the users’ efficacy in the second testing session. In addition, the app interface and navigation elements were implemented to fit different device characteristics, and this should serve to further differentiate their user experience. All evaluation sessions except one took place in a quiet room at a community center with a moderator (JM). Only one participant was visited at home. The study was performed in the Norwegian language. The moderator guided the participants through the testing procedure but did not intervene or disrupt the thinking process. Help was provided to participants only if they explicitly requested it, and only the essential amount of information to enable them to move on to the next task was provided.

All participants completed a demographic questionnaire that asked about their age, gender, education level, and previous experiences with mobile phone and tablet devices, and previous use of the Connect system. A tutorial of the Connect system and its functionalities was not given beforehand, since the initial assumption was that participants were already familiar with the system from prior use on the Web-based app on a personal computer. We also wanted to understand the nature of challenges involved in translating knowledge and procedures from one platform to another. The participants were offered pre-training exercises on the testing device prior to testing, with the goal of enabling participants with little to no experience with mobile touchscreen devices to gain a general understanding of the standard mobile system design and its navigational characteristics.

Throughout testing, each participant was asked to perform nine tasks of varying levels of complexity. The following tasks covered the full range of functions offered by the mobile app:

- Login/Logout to the app (Tasks 1 & 9).
- Reply to a message from the nurse (Tasks 2-3).
- Report the predefined set of health related symptoms (Task 4).
- Find self-management activities that can be performed to address new and previously reported health symptoms (Tasks 5-7).
- Share predefined text with other patients in forum (Task 8).

The participants were asked a set of follow-up semistructured questions following each group of tasks, with the goal of obtaining the participants’ immediate interpretation of a given task scenario and system design and to facilitate the elaboration of usability issues and increasing insight and design suggestions [51]. During this phase, the participants were encouraged to discuss the situations where they encountered problems or expressed concerns and then discuss the possible causes of the situation or possible design changes that could be implemented to address the identified issues.

After testing, the participants were asked to fill out the System Usability Scale, a standardized questionnaire used to assess participants’ perceptions of usability [52]. This robust and reliable scale consists of a 10-item questionnaire with each item rated on a 5-point Likert scale. The scores for each question were converted to a new number (odd questions score is calculated as scale position minus 2, and even-numbered questions are calculated as 5 minus the scale position), added together, and then multiplied by 2.5 to get the final score (ranging from 0 to 100).

At the end of testing, participants were asked follow-up questions about the full app design and its features, its usefulness, participants’ intention to use it, and barriers to the use of the mobile app in the future.

The participants were video-recorded and analyzed using Morae usability and analytic software (Techsmith). In addition, notes taken during observation of the sessions served to inform the analysis. During the analysis, we quantified and characterized a set of variables related to user performance. Specifically, we identified the number of requests for assistance, the types of errors participants made, and the time taken to complete the task. We also noted the feedback provided by participants in relation to their user experience. The list of identified events (request for help, errors, participant feedback, and moderator’s observation) was then used to assemble a list of usability issues. Each of the usability issues was categorized according to problem type and frequency of occurrence. The videos were reviewed and coded by the first author (JM). All identified issues were also reviewed by the second author (DK), and all differences were resolved through iterative viewings and discussions.

Each of the usability issues was mapped to usability heuristics for mobile devices to note any violations of usability principles. We employed usability heuristics for mobile computing as defined by Bertini et al [53]. The heuristics reflect a modification of Nielsen’s heuristics [54] with the goal to capture contextual factors in mobile computing. The usability heuristics are (1) visibility of the system status, (2) match between system and the real world, (3) consistency and mapping, (4) good ergonomic and minimalistic design, (5) ease of input, screen readability, and glanceability, (6) flexibility, efficiency of use, and personalization, (7) aesthetic, privacy, and social conventions, and (8) realistic error management.

All participant and moderator comments along with feedback were transcribed and analyzed using thematic analysis. Thematic analysis was used to code the data based on main categories identified in the user feedback and to gain a more structured set of user needs and expectations from this type of mobile system.

## Results

### Overview

In this section, we first present the results of the quantitative analysis of the usability study, including demographic data and usability scale questionnaire results. The results of semistructured interviews and observations captured during the testing follow. The results are categorized in groups based on the main topic and subtopics they address.

### Participant Characteristics

Table 1 lists the characteristics of the study participants. Of the 7 participants, 4 were women and 3 were men. The average age of the participants was 61 years (range 49-75). For education, 6 participants had completed grade school or high school education, and only one had higher education. All of the participants owned a private cell phone (5 had smartphones and 2 had regular cell phones), while only 3 participants owned a tablet. The most commonly used function on the Web version of the Connect system was the exchange of messages between patients and health care provider (7 participants), the diary where they note their private information (5 participants), and the forum where they can share information with other patients (4 participants). Among the least used functions were symptom assessment and self-management support (3 participants) and the information module (2 participants).

**Table 1:** Characteristics of the study participants.

Characteristics		n (%)
Age, median (range)		61 (49-75)
**Gender**		
	Male	3 (43)
	Female	4 (57)
**Education**		
	Elementary/high school	6 (86)
	University/college	1 (14)
**Own a phone**		
	Smartphone	5 (71)
	Regular cell phone	2 (29)
**Experience with the phone**		
	Low	0 (0)
	Medium	3 (43)
	High	4 (57)
**Own a tablet computer**		
	Yes	3 (43)
	No	4 (57)
**Experience with a tablet computer**		
	Low	2 (67)
	Medium	1 (33)
	High	0 (0)
**Use of Connect system features on the Web version**		
	Assessment module	2 (29)
	Symptom self-management support module	3 (43)
	Information module	2 (29)
	Messaging module	7 (100)
	Communication module	4 (57)
	Diary module	5 (71)

### Quantitative Results

The task completion times with numbers of errors participants made while performing tasks and number of times they requested assistance are provided in Table 2. Although the sample size was too small to do reliable tests of significance, it is apparent that the tasks were completed consistently faster on the tablet than on the mobile phone. However, as the standard deviations suggest, there was substantial variation between users. All the tasks required more time on the mobile phone except for the logout task (Task 9). Tasks related to performing symptoms assessment (Task 4) took the most time for all participants to complete. They also yielded the highest number of errors and requests for help. Perhaps the primary reason is that this task is more complex and requires the user to go through four different steps: (1) symptom selection, (2) assessment of symptom bother (eg, a rating of perception of pain or irritation), (3) assessment of importance to address the problem during meeting with health provider, and (4) review of assessment summary. Additionally, symptom assessment was one of the least used functions on the Web version of the Connect system. Since participants were not previously acquainted with this system feature, they encountered problems in understanding the organization of functionality and step sequence without an initial introduction. The task of asking a user to reply to a message from the nurse and attach a picture to the message also took more time for patients to perform, even though the messaging function is one of the most frequently used features on the Web version for all participants. The high completion time and number of errors and requests for help indicate possible usability issues in implementation of the new picture attachment option.

**Table 2:** Quantitative results (time and standard deviation, errors, and requests for help) for both mobile phone and tablet app across nine tasks.

	Time (SD), seconds		Errors, n		Requests for help, n	
	Mobile phone	Tablet	Mobile phone(n=4)	Tablet (n=5)	Mobile phone (n=4)	Tablet (n=5)
Task 1^a^	101.25 (60.7)	67.6 (29.6)	1	4	2	4
Task 2^b^	321.75 (120.2)	293.2 (58.2)	7	5	11	20
Task 3^b^	42.75 (37.3)	28.4 (21.1)	2	0	3	2
Task 4^c^	682.75 (186.4)	403 (81.3)	10	5	26	30
Task 5^d^	191.75 (88.7)	116.6 (48.9)	2	2	7	16
Task 6^d^	116.75 (40.4)	108.0 (41.2)	2	1	7	11
Task 7^d^	132.75 (85.4)	69.4 (77.94)	2	2	4	3
Task 8^e^	186.75 (80.6)	142.8 (32.5)	1	1	4	4
Task 9^f^	13.75 (8.5)	22 (4.9)	0	0	1	1
Sum			27	20	65	91

^a^Task 1: Login to the app.

^b^Tasks 2-3: Reply to a message from the nurse.

^c^Task 4: Report the predefined set of health related symptoms.

^d^Tasks 5-7: Find self-management activities that can be performed to address new and previously reported health symptoms.

^e^Task 8: Share predefined text with other patients in forum.

^f^Task 9: Logout.

The number of requests during testing was very high, with participants making more requests for help when using the tablet app. One explanation could be that a majority of them owned mobile phones and had prior experience with these types of devices, which resulted in increased levels of self-confidence and fewer requests for help. However, 4 patients did not own a tablet and of the 3 patients who owned a tablet, 2 had limited experience and needed routine guidance. Frequency of requests across tasks was highest for the symptom assessment and messaging module, which also required the most time for participants to complete (as previously discussed).

The number of errors participants made during testing was higher for the mobile phone app and, consistent with previous observations, was the highest for the symptom assessment and messaging module. Additionally, in the tablet app, participants made a greater number of errors while logging into the app. The errors in this task were related to the participant’s lack of experience with the use of the virtual keyboard (eg, they frequently used the wrong buttons on the keyboard or the navigation bar, which either hid or displayed the keyboard).

The average subjective usability ratings from the System Usability Scale questionnaire were 71.25 (SD 14.8) for the mobile phone app and 72.5 (SD 15.3) for the tablet app. On the System Usability Scale, 68 is considered an average score [55]. From the results, we can conclude that participants, on average, rated both apps as being slightly above average. However, from the patients’ individual ratings, we observed that 4 patients rated mobile apps rather high with scores over 80 and the other participants rated the mobile app as below average (lower than 68). This result demonstrated that patients had a variable experience in using the app. In addition, it was apparent that the app did not support the full range of their requirements.

### Qualitative Results

#### Overview

The thematic analysis of the interview transcript and users’ feedback during the usability testing process revealed two main themes: (1) mobile app user-friendliness, with subtopics of Mobile app design and functionality issues, Self-efficacy and training, and Ease of use, and (2) usefulness of the Connect system, with subtopics of Context dependent usefulness of system features, System usefulness and intention to use, and Integration of new features.

#### Connect Mobile App User-Friendliness

##### Mobile App Design and Functionality Issues

The quantitative and qualitative analysis of the usability testing results together with the qualitative analysis of interviews with participants revealed design and functionality issues of the Connect Mobile app that have a potential to influence its effective and efficient use. The testing revealed a total of 27 design issues with the apps (13 for mobile phone and 14 for tablet version) (see Tables 3 and 4). Each of the identified issues was mapped to source events (ie, errors, requests for help, participant’s concurrent feedback, and moderator observation) used to identify the issue. Additionally, each of the issues was categorized in one of eight usability heuristics for mobile devices defined by Bertini et al [53].

**Table 3:** Usability issues identified in the Connect app for mobile phones.

App module	Problem	Source (frequency)	Heuristic
General	Color consistency between mobile and Web app	Feedback (1)	3 – Consistency and mapping
	Navigation issue—support more advance options for expert users (eg, swiping screen)	Feedback (1)	3 – Consistency and mapping
Messaging	The option for adding a picture to the message difficult to find and use	Errors (1); Request for help (2)	5 – Ease of input, screen readability, and glanceability
	The feedback about attached image is difficult to find	Request for help (3)	5 – Ease of input, screen readability, and glanceability
	Problems writing/editing a message—support inputting text in horizontal mode	Feedback (2)	5 – Ease of input, screen readability, and glanceability
	Unnecessary popup	Request for help (2)	4 – Good ergonomic and minimalistic design
	Popup screen options not intuitive	Errors (1)	2 – Match between system and the real world
Symptom assessment	Collapsible set widgets not intuitive	Feedback (2); Errors (3); Request for help (12)	5 – Ease of input, screen readability, and glanceability
	Slider widgets not intuitive	Request for help (5)	5 – Ease of input, screen readability and glanceability
	Introduction screen not intuitive nor informative enough	Errors (1); Request for help (4)	2 – Match between system and the real world
	Symptom assessment values in the summary screen not intuitive	Request for help (1)	2 – Match between system and the real world
Symptom self-management support	Color of the bottom tab menu not intuitive	Errors (1); Request for help (3)	5 – Ease of input, screen readability and glanceability
	Option for removing item from the list misleading	Errors (1)	2 – Match between system and the real world

**Table 4:** Usability issues identified in the Connect app for tablets.

App module	Problem	Source (frequency)	Heuristic
General	Color contrast across platforms	Feedback (4); Observations (1)	5 – Ease of input, screen readability, and glanceability
	Navigation issue—provide support for hardware back option	Errors (4); Request for help (2)	3 – Consistency and mapping
	Small font size	Feedback (1)	5 – Ease of input, screen readability, and glanceability
	Navigation issue—provide support for more intuitive navigation buttons (eg, text in navigation buttons)	Feedback (1)	3 – Consistency and mapping
Messaging	The option for adding a picture to the message difficult to find and use	Errors (1); Help (3); Observations (1)	5 – Ease of input, screen readability, and glanceability
	Font size too small for screens with more information (message info, content of the message)	Feedback (2); Observations (3)	5 – Ease of input, screen readability, and glanceability
	The feedback about attached image is difficult to find	Requests for help (2)	5 – Ease of input, screen readability, and glanceability
	Popup screen options not intuitive	Requests for help (1); Observation (1)	2 – Match between system and the real world
	Problems writing/editing a message	Observations (1)	5 – Ease of input, screen readability, and glanceability
	Difficult to answer to the message if the original content is not shown	Observation (1)	2 – Match between system and the real world
Symptom assessment	Collapsible set widgets not intuitive	Feedback (1); Help (10)	5 – Ease of input, screen readability and glanceability
	Slider widgets not intuitive	Observations (2); Requests for help (2)	5 – Ease of input, screen readability, and glanceability
	Introduction screen not intuitive nor informative enough	Requests for help (1); Observations (1)	2 – Match between system and the real world
Symptom self-management support	Font size too small for screens with text describing advices	Feedback (3)	5 – Ease of input, screen readability, and glanceability

As suggested by the results of the quantitative analysis, the majority of identified usability issues were related to messaging (11) and symptom assessment (7) modules. In the messaging module, participants had the most problems with a new feature that allowed them to attach a picture to the message. The option for attaching a picture, placed at the bottom of screen as a link, was hard to find and hard to use (Multimedia Appendix 1). Additionally, when the picture was added to the message, the feedback to the user in the form of a thumbnail image at the bottom of the screen was not visible since the user could not see it without scrolling down the page. Some participants who tested the tablet app commented that they would prefer to have a larger font size for screens when there is more information, for example, a screen showing health related advices and activities (Multimedia Appendix 2). Additionally, we observed that some of the participants experienced problems with inputting information. They also suggested the need for introducing additional affordances, such as writing text in a horizontal mode and quoting the original text of the sender’s message when writing a reply.

For the symptom assessment module, the participants struggled the most with understanding the collapsible set widgets that are used to present different categories and subcategories of symptoms (Multimedia Appendix 3). The main issue related to collapsible set is that participants had problems differentiating between different levels of hierarchal organization provided by the widget functionality. Additionally, one participant misinterpreted the standard plus/minus icons that are used to indicate that the categories are opened or closed. Some of the participants had problems understanding how to use slider widgets to set level of bother and importance for specific symptom. In the introduction screen, most participants did not understand the meaning of the text and often confused images in the text with buttons (Multimedia Appendix 4). Additionally, general design issues not related to specific system functionality were identified, most of which related to providing more intuitive or advanced navigations. For example, one of the participants with extensive mobile device experience commented that she was accustomed to navigating between separate parts of the apps by performing a swiping motion on the screen. Additionally, participants often wanted to use a hardware back button (if it exists on the device), and one participant proposed using both text and icons on the navigation buttons on the tablet app to support easier recognition and recall of options.

Most of the identified issues (15 issues) were related to usability heuristics addressing ease of input, screen readability, and glanceability (Heuristic 5). Such issues indicate that throughout the study participants mainly struggled with options related to inputting data (such as previously mentioned issues for attaching picture or selecting symptoms and botherness and importance level), and previewing information on the device’s small screen (eg, organization of symptoms in collapsible sets, font size). Additionally, the high number of usability issues that violated Heuristic 2, describing mismatch between system and real world (7 issues), and Heuristic 3, describing inconsistency and wrong mapping (4 issues), showed that understanding and interaction with system features were additionally challenging in some parts of the apps (eg, meaning of icons and system feedback, support for advanced navigation options). It should be noted that we did not identify any violations to the following heuristics: visibility of system status of the mobile device (Heuristic 1), flexibility and personalization (Heuristic 6), aesthetic, privacy, and social conventions (Heuristic 7), and error management (Heuristic 8). Problems associated with Heuristic 7 were not likely to emerge given the nature of the tasks.

##### Self-Efficacy and Training

As discussed previously, participants often felt insecure about their actions and asked for help and confirmation before performing tasks (Table 2). This is partially attributable to their lack of experience with mobile devices and perhaps their age as well. All participants commented that they would need some time to learn and get used to the system and its features before they could start to use it regularly. Some indicated a need for training prior to use. A couple also noted that they often ask for help from people in their surroundings (eg, family, friends) when using new functions on mobile device:

I have a lot of grandchildren that are really helpful to me in understanding the system. But they do it a little too fast. Since they [already] know it. And that is exactly my problem. When they are trying to explain [things] to me, I do not manage to follow them.

Patient 5, Tablet

During the testing, a couple of participants commented that for a younger generation with more experience with technology, the mobile app would be easier to use and more widely accepted for everyday health management than for an older user group:

Fortunately I have a smartphone [with touch screen]... So I am used to [this type of systems]… If I were 69 years old, and had an older Nokia phone with regular keyboard, it would be more challenging to use touch [screen]...And also I am not afraid to press something wrong. And this would be more difficult for people that that are maybe a little fearful.

Patient 1, Mobile phone

By the end of testing, most of the participants had gained some experience with the system and mobile app features, which led to much faster task completion times and a reduced number of requests for help. Two of the participants even commented that they were very satisfied with how they performed the tasks. All of the participants agreed that by using the app regularly they would be better acquainted with its features and use it more efficiently:

I think I will be able to use [the app], but it will take time. These tasks…When it is new then there are a lot [of new things]. However, I did this in the Connect [Web app]. So I can find what I want now [in the system].

Patient 3, Tablet

##### Ease of Use

After testing, all participants commented that it was not hard to perform the tasks and they were able to find the right functions in the app menus. They concluded that the mobile app is simple to use, and one participant stated that it is much easier to perform and understand tasks on a mobile phone app than on the Web version. After testing the tablet version, the same patient additionally concluded that the tablet version is even easier to use compared to the mobile phone and Web apps.

Most of the participants commented that they did not want more functions on the mobile phone and tablet apps because they would be more complicated and harder to use. However, other participants suggested that supporting further functionalities on mobile app(s) could be useful for users with greater expertise in these emerging technologies.

#### Usefulness of the Connect System

##### Context Dependent Usefulness of System Features

As noted before, all participants in the study were involved in the larger study and had previously used the Connect system on a Web browser on a personal computer. The background questionnaire showed that the symptom assessment and self-management support and information modules were the least used functions on the Web app (Table 1). Most participants in this study reported that the main reason for not using the symptom assessment and self-management support modules was that they did not have the need for these functions in their phase of illness. They commented that these features would be better suited for patients who were recently diagnosed since they would be experiencing a range of new symptoms and have numerous emerging concerns related to their health condition. Only one participant said that she did not possess strong enough knowledge of the system, which influenced her use of the different system features:

I always said that is important to get access [to the Connect system] when you are in the treatment, when you are new cancer patient. When some time passes, then you usually go beyond this part (points to the screen with registration process). Then this [symptom assessment and self-management support functions] is not what it is important. There are other things that are a little more important. But for people that get cancer, and are new cancer patients, this [functions] is very important.

Patient 3, Tablet

In addition to their previous comments about how usefulness of system features are related to the phase of the illness, after testing the mobile app some of the participants concluded that the usefulness of the system features could also be influenced by different contexts of use such as terminal type. They commented that symptom assessment and self-management support features, which were not frequently used on the Web app, could be used on mobile devices to overcome limitations introduced by the mobile device’s characteristics. For example, since writing text is more difficult on a mobile device, symptom assessment functionality can be used for easier registration and management of health problems instead of the diary functionality. Additionally, the symptom self-management module can be used to find practical advice on how to address health issues and presents an alternative to writing messages to the nurse.

##### System Usefulness and Intention to Use

Regarding Connect system functionalities, the participants commented that Connect system provides a lot of quality information, which is obviously important for this type of system. One participant additionally commented that for her one of the main goals of the Connect system was to provide support for easier patient-provider communication and higher levels of cooperation between patients:

When I had acute problems I had to go to the doctor. But for more chronic problems, like thoughts, emotions, experiences, physical problems… And when I finally sat down and wrote and defined all my problems… I could relax and be home. And not have to go to the doctor, and get appointments as I did [before]. And I knew I will get the answer and that there is somebody there who heard me. And then you as a cancer patient do not feel so alone…And that’s where you can get support through SMS or system as Connect.

Patient 1, Mobile phone

It is not just the fact that health care personnel should be available and give you answer in 24 or 48 hours. But we should be help to each other… I think the overall goal of the system and its functionalities is the meeting point for cancer patients… So we can share experiences about our anxiety, treatment, diagnose… And they [the patients] can be of help to each other.

Patient 1, Mobile phone

All participants agreed that both phone and tablet versions of the app are useful and that they would use them in future. Some of the participants even commented that they previously thought about or tried accessing the Connect system over mobile device(s). Some of the participants also commented that if they could have the opportunity to use the Connect system on a mobile device, they probably would not need to use the Web version.

Most of the participants commented that they would use the same features on the Connect Mobile app as they currently use on the Web app, but they identified new contexts of use that were enabled by mobile terminal characteristics:

So I got the [notification] message on my mobile phone [that I received a new message in Connect system], and only when I came home in the afternoon could I log in [and read it]. But tablet or mobile phone people have them with them all the time so they can log in and see [the message] right away.

Patient 4, Tablet

##### Integration of New Features

In addition to testing standard features of the Connect system on the mobile app, we also investigated how they can be further expanded with new functions that better fit mobile terminals, such as tools for sending pictures to health care providers as attachments to messages. All participants agreed that this function would be very useful and that they would use it since adding a picture to email or text message is a commonly performed action on their own mobile devices. One participant additionally suggested that rich media (such as pictures and video) could be integrated with blog or forum modules to support easier sharing of different information between patients. The same patient commented that since she does not have a good memory as a result of her treatment, she frequently takes pictures of her medications with the camera on her phone to facilitate management and recall of the medication information during consultations with her health care provider.

## Discussion

### Design Recommendations

Mobile devices are ubiquitous tools in everyday life and are increasingly becoming part of the armaments for patients in their efforts to manage chronic disease. However, the available tools often do not support the needs of patients. There is a need to better understand obstacles in order to use these tools more productively and to fashion appropriate design solutions to better suit user needs. We identified a set of design issues in our findings that could also be used to further improve mobile app functions and design in health care contexts. From these issues we defined a set of general design recommendations that can be used when developing patient support mobile apps with similar design and functionality requirements. The design recommendations, grouped by the device type (mobile phone app, tablet app, or both), and usability heuristics are presented in Table 5.

**Table 5:** The design recommendations grouped by the device type and usability heuristics.

App type	Heuristic	Design recommendations	Useful design features
Mobile phone and tablet	5: Ease of input, screen readability, and glanceability	Make sure the contrast between colors is clearly noticeable across different devices types	Easy identifiable back option
		Most important options/buttons/feedback to users must be visible on the screen without scrolling	Support buttons with both icons and text
		Collapsible sets and slider widgets may be problematic to use and understand	
		Implement support for using templates when writing messages to avoid requirement for too much writing	
	3: Consistency and mapping	Use the same colors across both Web and mobile systems	Implementation of system features to resemble to standard mobile app(s) options
		Enable both software and hardware back buttons (if device supports it)	System features should resemble across all apps (Web, mobile phone, tablet)
	2: Match between system and the real world	Make sure images and icons used in the app do not resemble of buttons	
		Text and options in dialog boxes must be easy understandable and intuitive	
		Enable user to preview original message content when writing reply	
	4: Good ergonomic and minimalistic design	Avoid use of not required popups/dialogs	
Mobile phone	5: Ease of input, screen readability, and glanceability	Provide support for writing message in horizontal mode	
	6: Flexibility, efficiency of use, and personalization		Option to customize main menu features
	3: Consistency and mapping	Implement advanced navigation options (eg, swiping screens) for expert users	
Tablet	5: Ease of input, screen readability, and glanceability	Use bigger font size (compared to the app for mobile phones) and additionally allow users to adjust text size themselves by zooming on screens with much text	Organization of the content on the screen in the two areas: menu on the left side and the main content on the right side
	3: Consistency and mapping	Use both text and icons on buttons	

Results showed that when performing tasks, patients mostly relied on their prior experience with mobile devices and Web versions of the Connect system and used knowledge along with analogies of these more familiar domains in order to build robust mental models of less familiar domains on the Connect Mobile app. The patients usually made mistakes or requested help when interface elements were not shown in a way that matched their previous experience with similar systems and real world perception (Heuristic 2). Inconsistency in mapping of system functions and interactions to standard mobile systems and the Connect system’s design and functions (Heuristic 3) also contributed to incorrect mappings across different mental models and served to diminish task performance. For example, one patient commented that the use of consistent colors across apps and more advanced standard navigation options on mobile phone app (swiping screens) would help her to transfer knowledge from one context to the other. Most of participants during interviews commented that they perceived the system features they previously used in the Web version or on private mobile device(s) as easier, since they were able to recognize the same features across different apps and/or devices. The previous experience with the Connect Web app also influenced the effectiveness of task performance. Based on these results, we can conclude that use of some standard design rules (eg, colors, system icons, option names) across all apps (Web, mobile phone, tablet) can help users transfer their knowledge of the Connect system to different contexts. Also, the development of a native mobile app that could be used on patients’ private mobile devices, and adapting its features to resemble ones on the standard mobile devices (eg, organization of content, menus, navigation) can further support users to create more accurate mental models of the mobile app and its features while providing a better match between system features and the real world. This conclusion is consistent with findings from related research work that also underline the importance of developing mobile health care apps for use on patients’ private phones while complying with standard mobile design and functionality rules that patients are familiar with, rather than compelling users to learn to interact with additional mobile device(s) [23,33].

Due to the limited characteristics of mobile devices (eg, small screen, limited input capabilities), different issues influencing ease of input, screen readability, and glanceability (Heuristic 5) are identified. For example, participants did not always remember to scroll down, which caused them to miss some of the options that were not visible on the top of the page. Similarly, collapsible sets and slider widgets were problematic to understand and use. Due to both age and ongoing health treatments, several participants reported physical problems that limited their use of mobile devices (eg, swollen hands that introduced additional difficulties when navigating and using touch screen; memory problems). In the literature, different research projects have investigated how universal design rules and guidelines should be applied to support the development of mobile apps for users with special needs. For example, Kane et al proposed more general guidelines for developing mobile services for people with visual and motor disabilities, such as support for highly flexible interface customization to arbitrary settings, and dynamic adaptation of user interface to increase accessibility in different outdoor environments [56]. More specific design approaches have been created to facilitate interactions for people with specific needs, such as sliding fingers on the screen instead of tapping for people suffering from tremors [57] or using pens and edges on the screen for people with motor issues [58]. Participants in our study also gave us some suggestions on how they usually address these problems when using their private mobile devices and proposed how the Connect Mobile app could be adapted to be more suitable to their needs. For example, a couple of patients suggested that the horizontal view mode must be enabled when writing text on mobile phones since in this manner the buttons are bigger and easier to press. In menus, both icons and text should be used, since remembering meanings of just icons can be difficult. Additionally, different options can be used to facilitate inputting text in forms (eg, using template text, bigger font size of input fields).

Aside from the design issues and problems observed in the study, patients also identified navigation and design features that they found particularly useful. For example, on the tablet app, participants were very satisfied with organization of the content on the screen in the two areas (menu on the left side and the main content on the right side) since it provided a better overview of page content and required less action from the user (Heuristic 5).

The results of the study showed that it would be useful to have customization options that enable users to manually adjust the visibility of app modules on the main menu. This option could be used to preserve mobile app simplicity, which is identified as one of the most important app features. The customization option would be especially useful if the mobile app were to be expanded to support more system features. In general, the customization of system options is a regular feature of mobile apps (the app world in particular) that enable users to adjust the content and features of the app to better fit their needs. This finding is consistent with several other mHealth studies that underscore the importance of enabling user customization [7,33,59,60]. Of course, unbridled customization would lead to app inconsistency and result in possible user confusion, so it is necessary to strike a balance between consistency and flexibility [61].

### Connect System in the New Context of Use

The results from our study showed us patient needs for different system features on mobile phones and tablet devices, and how they differ from their Web app needs. Some patients said that they would use mainly the same functionalities on the mobile device as on the Web app, but also identified how the same features can be useful in the new contexts introduced by mobility of access terminal (eg, having the option to read the message from the health provider as soon as the SMS notification arrived, enabling access to the forum and blog features when traveling or away from home). Other patients identified features that they had not previously used on the Connect Web app as more useful on the mobile app. For example, since typing text was a demanding task for this patient group, some of them proposed using alternative system features such as menus that require selection from a predefined list (eg, using symptom assessment functionality for monitoring and reporting health symptoms from predefined lists instead of writing and describing symptoms in free text in the diary module). This is a classic problem in design that reflects the tension between flexibility/expressiveness and the need for standardization and structure [62]. Additionally, the symptom self-management support module can be used by patients to find advice and identify possible solutions related to reported symptoms instead of having to write messages to the nurse to solicit their advice. These results showed us how patients change and adapt their health management needs based on the current context, and how these new needs are influencing perception of usefulness and acceptability of different system features in the new contexts of use.

The results of this study were consistent with some of the common usage patterns for the current Web version of Connect system [41,45]. For example, this group of patients used the Web app more as a communication tool that enabled them to communicate with other patients and to provide each other with support, as well as to exchange information with their health care provider. In fact, one of the participants erroneously believed that the symptom assessment module generated a summary report that was automatically shared with his health care provider. Additionally, two other participants asked if it was possible to share lists of report problems in Connect with their health care provider. The feedback we gained during the study underlined the importance of system features that enable patients to involve health care personnel in health issues management and leverage socialization and sharing experience with other patients [7,40,41]. This is a prerequisite for effective shared decision making, which is especially important for cancer patients [63].

The participants reported that the acceptability of patient support systems and its features are influenced by the phase of illness. For example, system features that enable patients to self-manage their symptoms and health-related issues are particularly important for patients in the early phases of their illness and/or treatment. This is consistent with results from previous studies [31,40,45,64,65].

### Mobile App Usefulness and Directions for Further Development

All participants agreed that the Connect Mobile app is useful and that they would use it in the future for managing their health conditions. The results of the study support the notion that patient support systems for cancer patients, such as the Connect system, should be available across multiple modalities including Web and mobile devices. Integration of different mobile devices that provide the new context of use and advanced features are required to enable the full potential of patient support systems (such as personal health records and patient portals). Such systems would serve to increase use and accessibility for patients and promote shared decision making with health providers.

Participants also provided feedback on how different system features could be further developed and improved to support more efficient communication and collaboration. For example, using new information formats such as images in messages and blog modules can help patients to share their experience and health issues with others. The related research work on use of rich media as pictures [66,67], voice [33], and videos [68] in managing health conditions showed positive results and identified mobile devices as suitable tools to more quickly capture richer data, which was not previously possible using stationary computers. The results of our study are consistent with these findings and show patients’ preferences on how rich media can be integrated as part of different functions of the Connect system for both sharing and managing personal health information. Also, integrating symptom assessment modules with electronic health care records supports more efficient symptom monitoring and enhances shared decision making between patient and health care provider.

### User Training

The results of the study identified existing misunderstandings about Connect system functionalities showing that the patients’ mental models of the system do not completely correspond with the real system functions. For example, as reported previously, one patient used the symptom assessment module because he believed that this information was visible to his health care provider and complained that he never received any return communications. He did not understand that the reported symptom should be used for self-management and that he can use symptom self-management support module himself to find advice addressing previously reported symptoms. One other patient was reluctant to write and share thoughts and comments with other patients since she was concerned about privacy issues and did not realize that only her nickname (and not identifiable information) was shown to others in the discussion forum.

These misunderstandings suggest that better training for new users, including a more detailed explanation of the system functions, is necessary for both proper use of the system and its acceptance. This is especially important with older users with limited prior experience with technology [69,70]. This study is a precursor to a large-scale clinical trial of the Connect Mobile app. The findings of the study highlight the need for effective training to avoid possible mismatch between user mental models of the system and system. The fact that a majority of the participants became proficient during a 1-hour testing period showed us that the training period does not need to be very long, but it should focus on the system features that are more problematic and complex for potential users.

Some participants argued that the mobile technology is more suited for younger patients than for older users due to their limited experience with aspects of these devices such as the touchscreen display. However, even though most of the participants in this study were middle aged or older and not proficient users of mobile devices, all of them showed interest in using these apps to access the Connect system. This trend is also shown by some of the previous research, which demonstrated that older adults are also interested and capable of using emerging devices and advanced services for managing health care issues [71-73]. Additionally, the lower level of education of most participants did not influence the acceptance of the mobile app in our study, contrary to previous research findings [38].

### Limitations and Future Work

We recognize that the user group of 7 patients who were involved in this evaluation study constitutes a small sample and may not adequately represent the larger user population. However, the sample size is more or less consistent with general recommendations for usability testing that state that the majority of usability issues can be identified with smaller number of participants (eg, 5-7 participants) [74,75]. It provided us with valuable feedback about the current app design and identified significant usability issues that will be addressed in the iterative design process. Additionally, we believe that participants’ prior knowledge and experience with the Connect system enabled them to more readily assess the Connect mobile app’s usefulness, evaluate potential new contexts of use, and suggest new functions to augment the existing system.

Although we can learn a lot about the usability of a mobile app in a controlled setting, it is important to test it in real-world situations, which are highly variable [76]. In the future, and prior to the clinical trial, we are planning to organize a feasibility study (outside a laboratory context) to further identify implementation issues and context-related concerns about the system features and design.

Previously we noted that there is limited related research on how mobile devices can be used in the context of health care information systems for cancer patients. These patients have special needs and unique problems. Further work is needed to identify the primary factors and design issues influencing acceptability and usefulness of different system features of mobile health care information services. In our future research, we are planning to continue work on development of Connect Mobile app and investigate how apps for mobile phones and tablets can be designed and adjusted to best fit users’ needs in the new contexts of use. Some of the potential new app features have been identified during testing, as well as the need for further exploration of how we can add rich media to the Connect Mobile app.

### Conclusions

This work describes the results of our study of the design and functionality requirements for developing a mobile app to support cancer patients’ management of health-related symptoms and problems.

The study has shown the need for and potential of integrating mobile phones and tablets in patient support systems and identified design recommendations and useful features that can be applied during mobile app design and development processes. The results of this study demonstrated how potential use and acceptance of different patient support system features could be influenced not just by usefulness of specific functions but also by current context of use. The results from the study will be used in the iterative development of the Connect Mobile app and can also be used by other developers and researchers in the development, integration, and evaluation of mHealth apps and services that support cancer patients in managing their health-related issues and problems. mHealth is a burgeoning area of research and application. However, we are not yet at the point where system development is based on a stable paradigm that factors in a host of usability and idiosyncratic user needs. Nevertheless, it is becoming more apparent that even users who are typically considered to be disadvantaged, including older adults and those with lower levels of education, will not only accept the technology but also embrace it.

## Multimedia Appendix 1

App screen with the option for attaching a picture on the bottom of the page that was hard to find and use (in norwegian).

## Multimedia Appendix 2

App screen showing the small font size on pages containing lots of text (in Norwegian).

## Multimedia Appendix 3

App screen with the collapsible set widget that was problematic for participants to understand (in Norwegian).

## Multimedia Appendix 4

App screen with images that were confused as buttons (in Norwegian).

## Abbreviations

CSS: Cascading Style Sheet
e-ESAS: electronic Edmonton Symptom Assessment System
HTML: hypertext markup language
HTTP: hypertext transfer protocol
PDA: personal digital assistant
REST: representational state transfer
SMS: short-message service
UCD: user-centered design
WHOMS: Wireless Health Outcomes Monitoring System
