# Supplementary figures and images for: Supporting Cancer Patients in Illness Management: Usability Evaluation of a Mobile App
Source: JMIR Mhealth Uhealth. 2014 Aug 13;2(3):e33. doi: 10.2196/mhealth.3359 (PMC4147703; doi:10.2196/mhealth.3359)

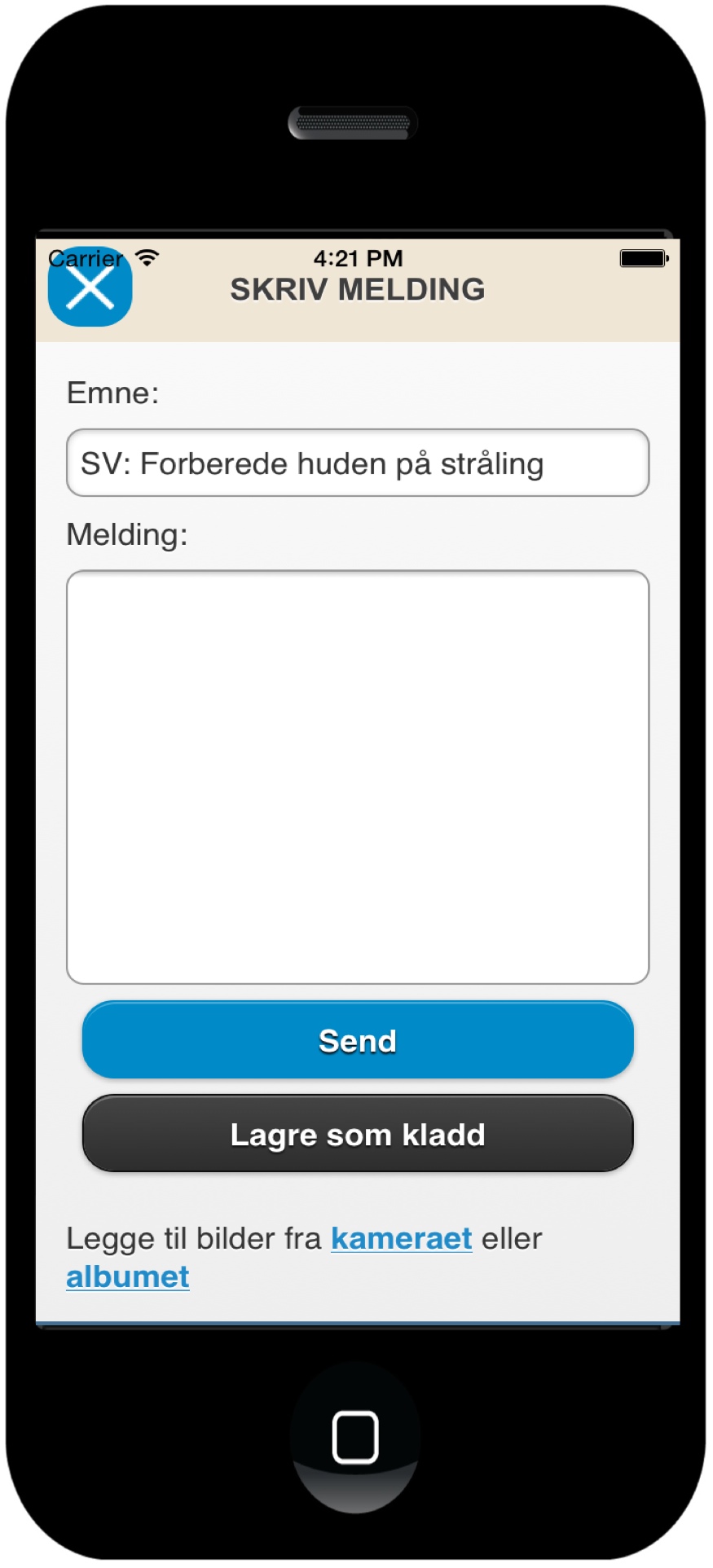

Supplement: Supplementary file 1 [file mhealth_v2i3e33_app1.jpg]

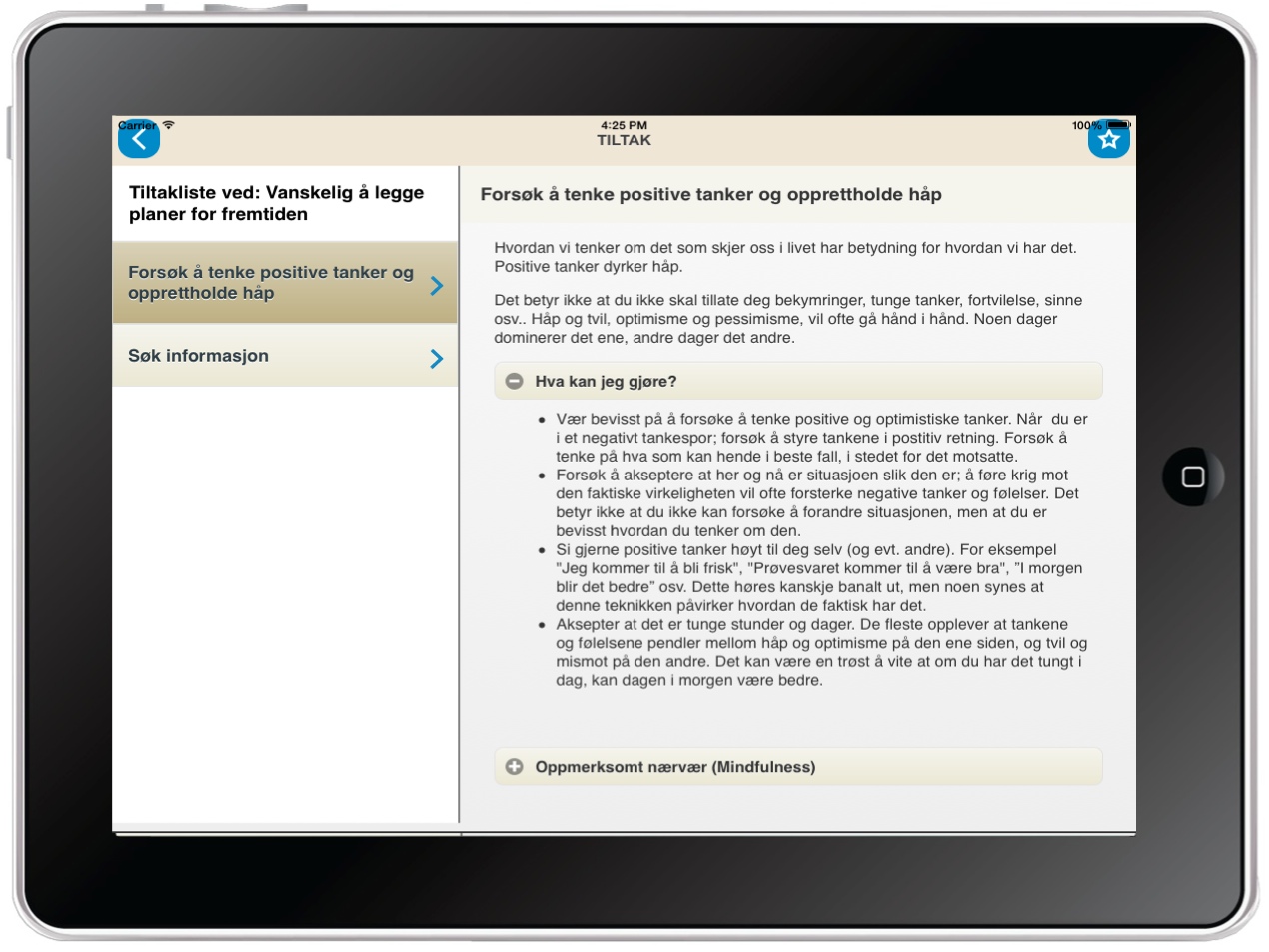

Supplement: Supplementary file 2 [file mhealth_v2i3e33_app2.jpg]

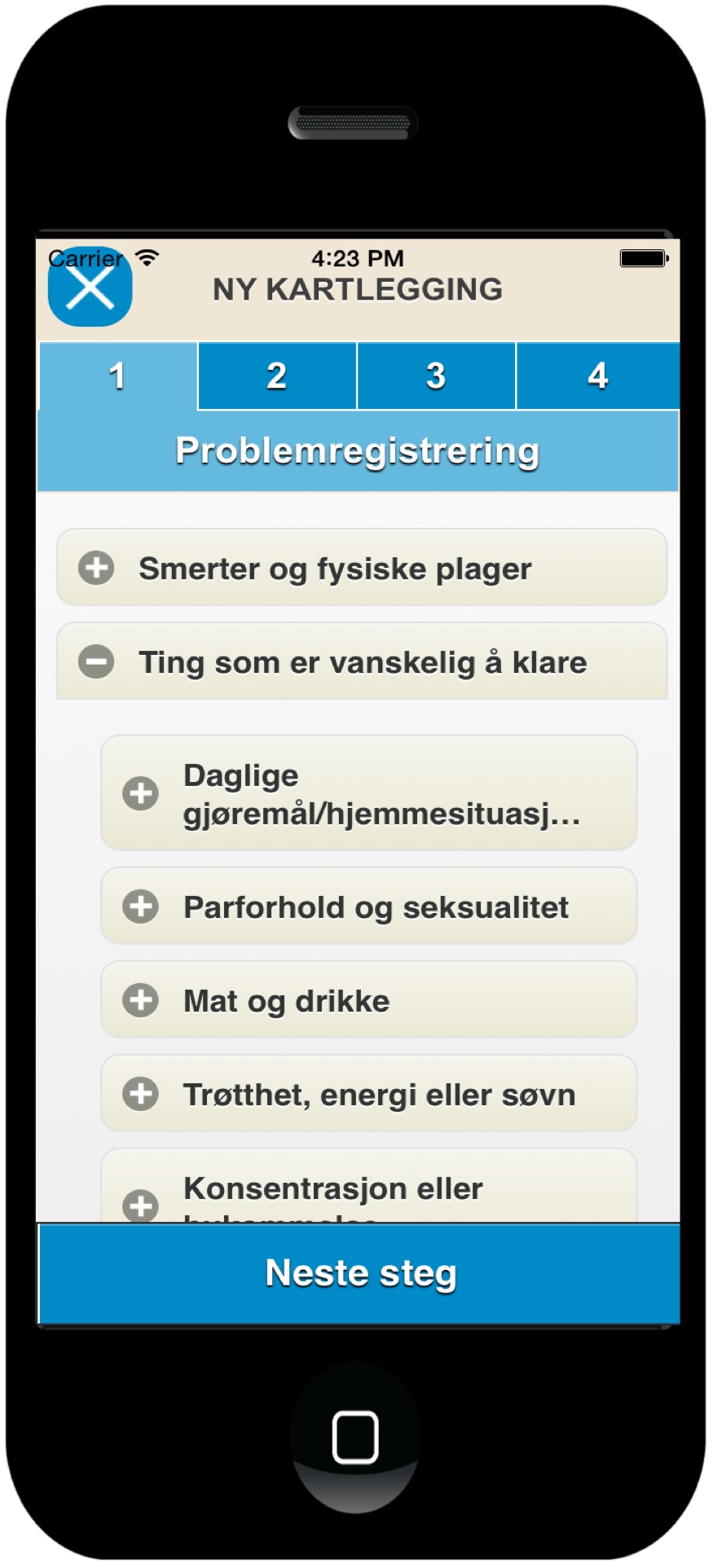

Supplement: Supplementary file 3 [file mhealth_v2i3e33_app3.jpg]

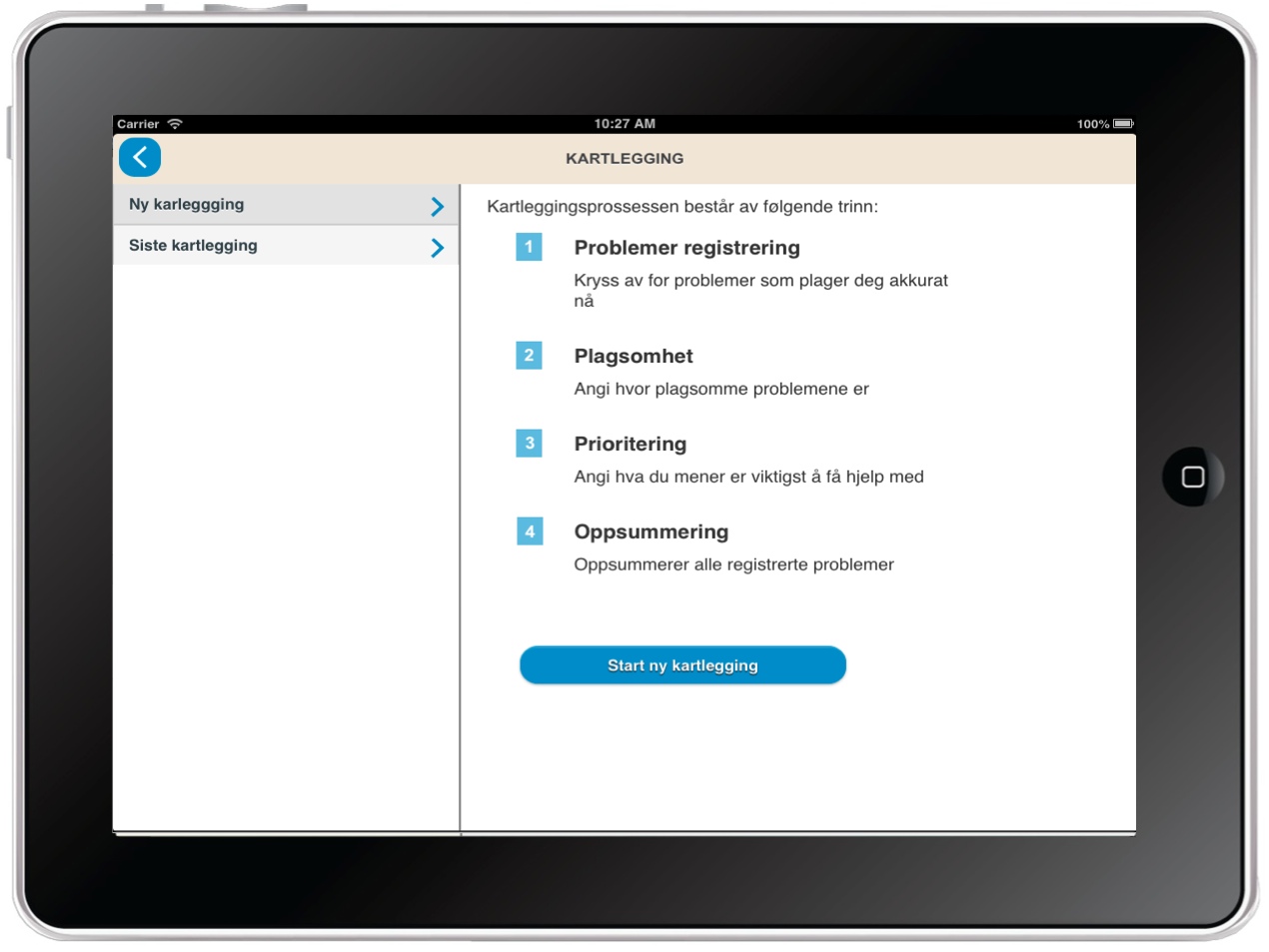

Supplement: Supplementary file 4 [file mhealth_v2i3e33_app4.jpg]
